# Screen time and developmental health: results from an early childhood study in Canada

**DOI:** 10.1186/s12889-022-12701-3

**Published:** 2022-02-15

**Authors:** Salima Kerai, Alisa Almas, Martin Guhn, Barry Forer, Eva Oberle

**Affiliations:** School of Population and Public Health, the Human Early Learning Partnership, The University of British Columbia, 2206 East Mall, BC V6T 1Z3 Vancouver, Canada

**Keywords:** Screen time, Developmental health, Early childhood, Health behaviors, Family income, Canada

## Abstract

**Background:**

Research has shown that longer hours of screen time are negatively associated with children’s healthy development. Whereas most research has focused on school-age children, less is known about this association in early childhood. To fill this gap, we examined the association between screen time and developmental health in preschool-aged children.

**Methods:**

This study draws from a data linkage on children (*N* = 2983; Mean age = 5.2, *SD* = 0.3 years, 51% male) in British Columbia (BC), Canada, who entered Kindergarten in public elementary schools in 2019. Parent reports on children’s screen time, health behaviors, demographics, and family income collected upon kindergarten entry (09/2019), were linked to teacher reports on children’s developmental health, collected halfway through the school year (02/2020). Screen time was assessed with the Childhood Experiences Questionnaire. Developmental vulnerability versus developmental health in five domains (physical, social, emotional, language and cognition, and communication skills) was measured with the Early Development Instrument.

**Results:**

Logistic regression analyses using generalized estimating equation showed that children with more than one hour of daily screen time were more likely to be vulnerable in all five developmental health domains: physical health and wellbeing (odds ratio [*OR*] =1.41; 95% confidence interval [CI], 0.99 - 2.0; *p=*0.058), social competence (*OR=*1.60; 95% CI, 1.16 – 2.2; *p=*0.004), emotional maturity (*OR=*1.29; 95% CI, 0.96 - 1.73; *p=*0.097), language and cognitive development (*OR=*1.81; 95% CI, 1.19 - 2.74; *p=*0.006) and communication skills (*OR=*1.60; 95% CI, 1.1 – 2.34; *p=*0.015) compared to children reporting up to one hour of screen time/day. An interaction effect between income and screen time on developmental health outcomes was non-significant. Results were adjusted for child demographics, family income, and other health behaviors.

**Conclusions:**

Daily screen time that exceeds the recommended one-hour limit for young children, as suggested by the Canadian 24-h Movement Guidelines for Children and Youth (Tremblay et al. BMC Public Health. 17:874, 2017; Tremblay J Physical Activity Health. 17:92–5, 2020) is negatively associated with developmental health outcomes in early childhood. Screen-based activities should thus be limited for young children. Future research needs to examine the underlying mechanisms through which screen time is linked to developmental vulnerabilities.

**Supplementary Information:**

The online version contains supplementary material available at 10.1186/s12889-022-12701-3.

## Background

In the past two decades, screen time (e.g., watching television, playing virtual games) has almost doubled among young children [1, 2]. Screens have a ubiquitous presence in children’s day-to-day lives, are easy to access, and serve as a frequent platform for activity and entertainment [3]. While screen use can be beneficial for children to some extent (e.g., participation in educational programs) [4], prolonged screen use in childhood has been linked to negative developmental health outcomes including obesity [5], behavior problems [6], emotion regulation problems [7], speech delays [8], lower executive functioning [9] and academic problems [10]. It has further been suggested that time spent in screen-based activities displaces free and active play in childhood to some extent, interferes with social interactions (e.g., with family and peers) and impacts sleep duration and quality [9, 11, 12]. Some research suggests that screen time may be particularly concerning for children from low income families. In a study with *N*=803 pre-school aged children, lower income was related to more time spent watching television; the association between watching television and lower cognition functioning and problem-solving skills was moderated by income and significantly stronger for children from low income families [13].

In response to the public health concerns resulting from increases in screen time, Canadian and international researchers, non-government organizations (e.g., ParticipACTION), and policy makers developed the 24-H Movement Guidelines for the Early Years in 2017, to encourage and promote optimal health behaviors in early childhood [14, 15]. The guidelines recommend that children under the age of five limit screen time to 1 h per day. Further, the guidelines recommend that children under the age of five be physically active for at least 180 min per day and get at least 10 h of sleep in a 24-h period. A recent study with a nationally representative sample of pre-school aged children in Canada found that while a majority of children met the recommendations specified by the 24-h guidelines for physical activity (61.8%) and sleep duration (83.9%), only a quarter of children met the recommended 1 h screen time limit per day [16].

The goal of this study was to examine whether meeting versus exceeding daily screen time recommendations in early childhood (i.e., >1 h/day) was associated with developmental vulnerability in social, emotional, cognitive, and physical domains, taking into account other important health behaviors (i.e., physical activity, sleep) and children’s demographic backgrounds (sex, family income, ethnicity, living in rural versus urban communities living).

This study addresses several gaps in research. Research to date has predominately examined screen time in older children [17–19] and less is known about the association between screen time and developmental health in early childhood. Further, few studies on screen time integrate other important health behaviors (e.g., physical activity, sleep) as well as contextual variables that are associated with children’s developmental health (e.g., family income, ethnic background, rural versus urban living). Whereas a majority of research has examined screen time in relation to physical health, such as childhood obesity [5], there is a dearth of research that takes a whole-child approach and incorporates a broader range of developmental health indicators in early childhood (i.e., physical, social, emotional, and cognitive health, and communication skills).

The present study draws from a large database of parent-reported child health behaviors and demographics collected upon Kindergarten entry, linked to teacher reports on children’s developmental health 6 months later. Based on previous research, we hypothesized that children who exceeded the daily recommended screen time limits were more likely to be vulnerable in social, emotional, physical and cognitive development, after adjusting for the effects of family income, child and family demographics, and children’s sleep and physical activity levels. Given that some research has shown differences in screen time among preschool-aged children by family income [13], we also examined whether there were differences in children exceeding screen time recommendations based on family income, and whether the association between screen time and developmental health was moderated by family income.

## Methods

### Participants

This study followed the Strengthening the Reporting of Observational Studies in Epidemiology (STROBE) reporting guidelines [20]. Participants were *N* = 2983 children in British Columbia (BC), Canada, who had participated in the Childhood Experiences Questionnaire (CHEQ; caregiver-report) Project and in the Early Development Instrument (EDI); teacher report) project in the 2019/20 school year. Primary caregivers’ responses on the CHEQ, collected in September 2019 [21] were linked to teachers’ responses on the EDI, collected in February 2020 [22]. The majority of the primary caregivers were parents: mothers (*N* = 2421, 81.2%) and fathers (*N* = 427, 14.3%). The CHEQ measures child and family demographics and children’s health behaviors at Kindergarten entry; the EDI measures children’s developmental health midway through the Kindergarten year. Detailed information on sampling for school districts for the CHEQ has been provided elsewhere [23].

Children in the sample came from 144 schools within nine school districts in BC (there are a total of 60 school districts and 1038 public elementary schools in BC) [23]. The mean age of children was 5.2, *SD* = 0.3 years at the time of the CHEQ implementation and 5.6, *SD* = 0.3 at the time of the EDI implementation. The average participation rate for the CHEQ across school districts was 57%. The CHEQ-EDI linkage rate was 98.3%. Individual child-level data on the CHEQ were linked to their EDI survey by Population Data BC via unique identifiers (i.e. Personal Education Numbers) [24]. Data were accessed via a secure server at Population Data BC [25].

### Measures

Information on all explanatory variables (i.e., screen time, sleep, physical activity ethnicity, sex, annual household income) was obtained from the CHEQ. Screen time was the main exposure variable of interest. Sleep, physical activity, family ethnicity, child sex, family annual household income, and rural versus urban community were included as covariates based on their role as potential confounders in predicting developmental health [17, 26–29]. Information on outcome variables (i.e., physical health/wellbeing, social competence, emotional maturity, language and cognitive development, communication skills) was obtained from the EDI. Descriptive statistics for all variables can be found in Table 1.

**Table 1:** Descriptive Statistics on Demographics and Health Behaviors Among Preschool-Aged Children in British Columbia, Canada

	Variables	Total sample *N* (%)
Explanatory Variables on the CHEQ		
Screen time	> 1 h	1183 (39.7%)
	≤ 1 h	1734 (58.1%)
	Missing	66 (2.2%)
Sex	Male	1558 (52.2%)
	Female	1425 (47.8%)
Ethnicity	European origins	1602 (53.7%)
	Non-Euro. origins^a^	1048 (35.1%)
	Missing	333 (11.2%)
Annual family income	< $75,000	826 (27.7%)
	≥ $75,000	1640 (55.0%)
	Missing	517 (17.3%)
Population Centre	Urban	1596 (53.5%)
	Small to medium	1387 (46.5%)
Physical activity	Nonparticipant	526 (17.6%)
	Participant	2416 (81.0%)
	Missing	41 (1.4%)
Duration of sleep	< 10 h	303 (10.2%)
	≥ 10 h	2616 (87.7%)
	Missing	64 (2.1%)
Dependent Variables on the EDI		
Physical health and wellbeing	Not vulnerable	2521 (84.5%)
	Vulnerable	319 (10.7%)
	Missing	143 (4.8%)
Social competence	Not vulnerable	2460 (82.5%)
	Vulnerable	377 (12.6%)
	Missing	146 (4.9%)
Emotional maturity	Not vulnerable	2408 (80.7%)
	Vulnerable	427 (14.3%)
	Missing	148 (5.0%)
Language and cognitive development	Not vulnerable	2580 (86.5%)
	Vulnerable	244 (8.2%)
	Missing	159 (5.3%)
Communication skills	Not vulnerable	2544 (85.3%)
	Vulnerable	295 (9.9%)
	Missing	144 (4.8%)

*Note*. *N* = 2983. ^a^ Non- European origins include families with Indigenous, East Asian, South East Asian, South Asian, Latin America, Middle East, African background

### Screen time

Screen time was assessed by asking parents: “Think about the past 6 months, on average, how much time per day did your child use an electronic device like a tablet, smartphone, TV or computer (alone)?”. Response options were 1 = None; 2 = < 15 min; 3 = 15 min to 1 h; 4 = 1-2 h; 5 = > 2 h. Based on the 24-hour movement guidelines [14] for the early years, screen time was categorized into 0 (≤ 1 h) and 1 (>1 h).

### Physical activity

Physical activity was assessed by asking parents “In the last 6 months, about how many times per week did your child take part in energetic physical activity while participating in organized activities (for example, swimming lessons or gymnastics lessons)?” Response options were 1 = Never; 2 = Once a week or less; 3 = 2-3 times week; 4 = 4-5 times a week; 5 = 6-7 times a week. For subsequent analyses, responses were grouped into 1 = participant (participation at least once per week) and 0 = non-participant (never participated). Distinguishing between participants and nonparticipants is a commonly used indicator for preschool aged children’s involvement in organized physical activity in absence of objective record of number of minutes spent in a day [30].

### Sleep

Sleep was assessed by asking parents “How many hours does your child usually sleep in a 24-h period (Combining night time sleep and naps)?” In response parents indicated number of hours. Based on the 24-h movement guidelines [14], responses were grouped into 1, indicating optimal sleep duration (i.e., ≥ 10 h) and 0, indicating less sleep than recommended (i.e., <10 h).

### Demographics

Families indicated their ethnicity by endorsing one or more of nine ethnic categories they were presented with. The category indicating European origins was checked by 53% of families. A binary ethnicity variable was therefore created for subsequent analyses: 0 = European origins; 1 = Non-European origins. Income was assessed by asking parents to indicate their best estimate of their family’s overall household income in the previous year before taxes: 1 = under $20,000; 2 = $20,000 to $49,999; 3 = $50,000 to $74,999; 4 = $75,000 to $99,999; 5 = $100,000 to $149,999; 6 = $150,000 to $199,999; 7 = $200,000 or more. For further analyses, we created a binary variable indicating whether household income was less than $75,000 versus $75,000 or more. This cut-off was determined based on the 2019 living wages for families of two parent household in Metro Vancouver where both parents were full-time ($19.50/hour, resulting in approximately $75,000 before taxes) [31]. Statistics Canada’s criteria were used to categorize school districts into urban (i.e. population of 100,000 and more) and rural (small and medium population centers i.e., <100,000) [32].

### Developmental health

Children’s developmental health was assessed in five domains: physical health and wellbeing (13 items); social competence (26 items); emotional maturity (30 items); language and cognitive development (26 items); and communication skills (8 items). Responses on these domains were assessed on 2- or 3- point Likert scales (i.e., yes, no, don’t know; very true, sometimes or somewhat true, never or not true, don’t know). In alignment with previous research with the EDI [33], all responses on binary items were coded 0 or 10 and 3-point Likert-scale items were coded as 0, 5, and 10. All items contained an additional response option, *don’t know* (coded 99), which was not included in the statistical analyses. For every item, 10 designates the highest (i.e., most positive, most developmentally desirable) score. For every domain, the average score was calculated for each child, ranging from 0 to 10. Subsequently, children’s domain specific scores were converted into a dichotomous measure of vulnerability (0 = not vulnerable; 1 = vulnerable). As in previous research with the EDI, vulnerability for a given developmental domain was indicated by a child’s score being below a domain-specific cut-off score that marked the bottom 10th percentile in the normative distribution of kindergarten children, based on the first cycle of data collected in BC [29, 34, 35]. The scoring procedure for the EDI was developed by the instrument’s authors in collaboration with educators and knowledge users [29, 34, 35]. Previous research has supported the use of vulnerability scores in research with the EDI [36, 37]. The EDI’s validity has been supported in previous research, and good interrater reliability has been established [38–40]. In the present study, the internal consistency was satisfactory for all domains (Cronbach’s alphas > 0.86), except for physical health (Cronbach’s alpha = 0.45). Regarding the interrelatedness of outcome domains, correlations were mostly small to moderate ranging from 0.32 (emotional maturity and language development) to 0.48 (language development and communication), except for a large correlation of 0.66 between social competence and emotional maturity.

### Statistical analyses

Exploratory and bivariate analyses were conducted among all explanatory and dependent variables. To examine the differences in children exceeding screen time recommendations based on family income, Pearson’s Chi-square test was used [41]. Next, to examine the association between screen time and the five developmental health outcomes, five logistic regression models with generalizing estimating equations (GEE) were built (‘xtlogit’ command with ‘population average’ option in STATA). The GEE was used as estimators of variance, employing an exchangeable correlation structure to account for clustering in the data (i.e., children nested within schools) [42, 43]. School ID was used as a cluster variable. The school-level variability on developmental health outcomes ranged from ICC = 0.12 to ICC = 0.14.

Of 2,983 children in the linked sample, 853 (28.6%) had missing values on one or more variables; 165 (5.53%) had missing information on at least one developmental health domain (see Table 1). Based on bivariate analyses with missing indicator variable, information was presumed to be ‘missing at random’ (MAR) and multiple imputation (MI) using chained equation approach was used to impute missing values [44]. The process was repeated m=28 times [45, 46]. At the final stage, following Rubin’s rules, [47] parameter estimates were pooled. Imputed values for outcome variables were excluded post imputation under ‘multiple imputation then deletion’ (MID) approach [48]. As a result, 2818 (94.5%) children with complete information on outcome variables were included in the GEE analyses.

In building the multivariable models, all the covariates were entered simultaneously into the model based on their role as potential confounder and bivariate association with the outcome [17, 26–29]. Unadjusted and adjusted odds ratios (*ORs*) and corresponding 95% confidence interval (*CIs*) were reported for each developmental health outcome. A two-way interaction between screen time and income was explored using analysis of variance (ANOVA). To support the stability of the findings, sensitivity analyses were conducted, using linear regression models predicting scores on the five developmental outcomes in their continuous, non-dichotomized form. A sensitivity analysis was also performed on the sub-sample of children with complete data before the imputation of missing values (*N* = 2130) to examine whether results were comparable. All analyses were completed using STATA version 16 [49].

## Results

Family income was negatively related to screen time (χ^2^ = 25.8, df = 1, *p* < 0.001); children in families with lower annual household income (i.e., <$75,000) were significantly more likely to have high levels of screen time compared to children in higher income families (i.e., ≥$75,000). Specifically, among children from lower income families, 46.6% had > 1 h of screen time per day, whereas 53.4% had ≤ 1 h of screen time per day; among children from higher income families, 35.7% had > 1 h of screen time per day and 64.2% had ≤ 1 h of screen time per day. Table 2 shows the results of bivariate analyses on the association between explanatory variables on the CHEQ and dependent variables on the EDI.

**Table 2:** Bivariate Relationships Between Explanatory Variables and Vulnerability (Vulnerable vs. Not Vulnerable) on Five Developmental Health Domains Among Preschool-Aged Children in British Columbia, Canada, using Logistic Regression Model Reporting Crude Odds Ratios And 95% Confidence Intervals

	Physical health and wellbeing	Social competence	Emotional maturity	Language and cognitive development	Communication skills
	*OR* (95% CI)	*OR* (95% CI)	*OR* (95% CI)	*OR* (95% CI)	*OR* (95% CI)
Screen time					
> 1 h	1.86*** (1.46 - 2.37)	1.78*** (1.43 - 2.23)	1.62*** (1.31 - 1.99)	1.93*** (1.47 - 2.52)	1.74*** (1.35 - 2.23)
≤ 1 h	Reference				
Sex					
Male	1.8*** (1.4 - 2.3)	2.85*** (2.24 - 3.63)	3.13*** (2.48 - 3.95)	1.4* (1.07 - 1.83)	1.93*** (1.49 - 2.5)
Female	Reference				
Ethnicity					
European origins	0.91 (0.7 - 1.18)	0.75* (0.59 - 0.94)	0.11 (1.16 - 1.16)	0.61** (0.45 - 0.82)	0.4*** (0.3 - 0.53)
Not Euro. origins	Reference				
Annual household income					
< $75,000	2.2*** (1.68 - 2.89)	2.06*** (1.61 - 2.64)	1.77*** (1.41 - 2.23)	2.81*** (2.09 - 3.76)	2.26*** (1.66 - 3.08)
≥ $75,000	Reference				
Population Centre					
Urban	0.84 (0.63 - 1.12)	1.01 (0.76 - 1.35)	0.84 (0.63 - 1.11)	0.81 (0.6 - 1.08)	1.43* (1.07 - 1.9)
Small to medium	Reference				
Physical activity					
Nonparticipant	2.79*** (2.14 - 3.65)	2.14*** (1.66 - 2.75)	1.88*** (1.47 - 2.41)	2.73*** (2.03 - 3.65)	2.65*** (2.01 - 3.5)
Participant	Reference				
Sleep					
< 10 h	1.68** (1.19 - 2.38)	1.79*** (1.31 - 2.46)	1.88*** (1.39 - 2.53)	2.21*** (1.54 - 3.16)	2.12*** (1.51 - 2.97)
≥ 10 h	Reference				

*Notes. N* = 2,818. ^***^p < 0.001. ^**^p < 0.01. ^*^p < 0.05. For each of the five developmental health domains, *vulnerable* is coded as 1 and *not vulnerable* is coded as 0

The results of bivariate analyses suggest that children with screen time of > 1 h per day were more likely than children with ≤ 1 h per day to be developmentally vulnerable on all domains of developmental health. Furthermore, boys were more likely to be vulnerable than girls; children living in families of non-European origins were more likely to be vulnerable than those of European origins; children living in families with annual family income <75,000 were more likely to be vulnerable than children in families with annual family income ≥ 75,000; children who did not participate in structured physical activity were more likely to be vulnerable compared to participants; children with <10 h of sleep were more likely to be vulnerable compared to children with ≥ 10 h of sleep on all developmental domains.

Results of multivariable analyses for the association between screen time and developmental health among children are reported in Table 3. The adjusted odds ratio (OR) represents the average odds of being vulnerable on a developmental domain for the preschool children in our study with screen time of > 1 h per day compared to those having screen time of ≤ 1 h per day. Accordingly, children with screen time of > 1 h per day compared to screen time of ≤ 1 h in a day were 41% more likely to be vulnerable regarding their physical health and wellbeing (*OR =* 1.41; 95% CI 0.99 - 2.0; *p* = 0.058), 60% more likely to be vulnerable regarding their social competence (*OR =* 1.60; 95% CI 1.16 – 2.2; *p* = 0.004), 29% more likely to be vulnerable regarding their emotional maturity (*OR =* 1.29; 95% CI 0.96 - 1.73; *p* = 0.097), 81% more likely to be vulnerable regarding their language and cognitive development (*OR =* 1.81; 95% CI 1.19 - 2.74; *p* = 0.006) and 60% more likely to be vulnerable regarding their communication skills (*OR =* 1.60; 95% CI 1.1 – 2.34; *p =* 0.015). Estimates were adjusted for child sex, family income, ethnicity, rural/urban community living, sleep, and physical activity. There was no significant interaction between income and screen time in relation to any of the developmental outcomes. Note that when removing the non-significant interaction term from the models, the marginally significant effects of screen time on physical health and wellbeing, and on emotional maturity changed to statistical significance (physical health and wellbeing: *OR =* 1.58; 95% CI 1.23 - 2.04); *p* < 0.001; emotional maturity: *OR =* 1.39; 95% CI 1.12 - 1.73); *p =* 0.003.

**Table 3:** Multivariable Logistic Regression Model Reporting Adjusted Odds Ratios And 95% Confidence Intervals for Relationship Between Screen Time and Vulnerability (Vulnerable vs. Not Vulnerable) on Five Developmental Health Domains Among Preschool-Aged Children in British Columbia, Canada

	Physical health and wellbeing	Social competence	Emotional maturity	Language and cognitive development	Communication skills
	*OR* (95% CI)	*OR* (95% CI)	*OR* (95% CI)	*OR* (95% CI)	*OR* (95% CI)
Screen time					
> 1 h	1.41^† 1^ (0.99 - 2)	1.60** (1.16 - 2.2)	1.29^† 2^ (0.96 - 1.73)	1.81** (1.19 - 2.74)	1.60* (1.1 - 2.34)
≤ 1 h	Reference				
Sex					
Male	1.73*** (1.34 - 2.23)	2.83*** (2.21 - 3.63)	3.13*** (2.47 - 3.96)	1.36* (1.03 - 1.79)	1.95*** (1.49 - 2.55)
Female	Reference				
Ethnicity					
European origins	1.12 (0.84 - 1.49)	0.88 (0.68 - 1.13)	1.08 (0.84 - 1.38)	0.77 (0.56 - 1.06)	0.49*** (0.36 - 0.66)
Not Euro. origins	Reference				
Annual household income					
< $75,000	1.55* (1.03 - 2.31)	1.89** (1.32 - 2.7)	1.42* (1.02 - 1.98)	2.42*** (1.56 - 3.77)	1.98** (1.27 - 3.1)
≥ $75,000	Reference				
Population Centre					
Urban	0.92 (0.67 - 1.27)	1.02 (0.77 - 1.36)	0.86 (0.66 - 1.14)	0.83 (0.62 - 1.11)	1.42* (1.07 - 1.88)
Small to medium	Reference				
Physical activity					
Nonparticipant	2.2*** (1.66 - 2.92)	1.68*** (1.28 - 2.2)	1.52** (1.17 - 1.99)	1.99*** (1.46 - 2.7)	2.12*** (1.57 - 2.87)
Participant	Reference				
Sleep					
< 10 h	1.43^†^ (0.99 - 2.05)	1.51* (1.08 - 2.12)	1.71** (1.25 - 2.36)	1.69** (1.15 - 2.48)	1.63** (1.14 - 2.33)
≥ 10 h	Reference				
Screen time X Income	1.31 (0.78 - 2.19)	0.87 (0.53 - 1.4)	1.21 (0.76 - 1.95)	0.77 (0.43 - 1.38)	0.72 (0.4 - 1.28)

*Notes. N* = 2,818. ^***^p < 0.001. ^**^p < 0.01. ^*^p < 0.05. For each of the five developmental health domains, *vulnerable* is coded as 1 and *not vulnerable* is coded as 0.

^†^ indicates marginal significance at *p <* 0.109

^1^ When the interaction term is removed from the model, OR 1.58; 95% CI 1.23 - 2.04); *p* < 0.001

^2^ When the interaction term is removed from the model, OR 1.39; 95% CI 1.12 - 1.73); *p* < 0.01

Sensitivity analyses based on the sub-sample of children with complete data supported the stability of our findings (see Table S1). Similarly, sensitivity analyses using linear regression models showed that the direction and statistical significance of all results were consistent with those obtained from logistic regression analyses (see Table S2).

## Discussion

Our findings show that screen use that exceeds the recommended daily amount in the early years is associated with developmental vulnerability. Specifically, more than one hour of screen time per day was positively associated with vulnerability in physical, social, emotional, and cognitive developmental health domains. These associations were statistically significant over and above children’s further health behaviors – physical activity and sleep, as well as income and other demographics. Our results substantiate the findings from previous research that have shown a negative link between screen time and physical [50], social-emotional [51] and mental health [7] outcomes among preschool-aged children. They further extend past research on screen time and early child development by taking a whole-child approach that incorporates multiple domains of developmental health (i.e., physical, social, emotional, cognitive), while also taking into account multiple health behaviors and child/family demographics.

In the present study, 39.7% of children engaged in screen time for more than one hour per day. Evidence indicates that screen use appears to be increasing for Canadian children under five [2]. High levels of screen time were more common in males, children with non-European ethnic backgrounds, and children with family income below $75,000 in our study. These findings are in line with previous research [52] and point to social disparities in screen time. This is an important finding because recent research with young children in several European countries has found that children experiencing socio-economic vulnerability – including low family income – spent more time in screen-based activities [53]. Several explanations have been offered for why children from lower income families tend to have more screen time. For example, for parents in lower SES families child care is difficult to afford and they often have other competing demands (e.g., work, other caregiving responsibilities) while taking care of younger children [13, 54]. In contrast to a previous study (13), family income did not moderate the relationship between screen time and developmental outcomes in our study.

High levels of screen time during the early years can be potentially detrimental for achieving developmental milestones, especially school readiness. While causality cannot be implied in this study, longer screen time can interfere with positive and health-promoting experiences such as physical activity, social contact with peers and family, and good sleep hygiene [12]. Moreover, high levels of screen time are of concern for developmental health because it tends to displace free play-based and leisure activities that enhance cognitive and social-emotional skills that are key in promoting Kindergarten readiness [11, 55]. More research is needed to examine the underlying mechanisms and pathways through which screen time is linked to developmental vulnerabilities.

### Limitations

Since schools’ and parents’ participation in the study was voluntary, non-response bias from the schools that didn’t participate in the survey and parents who didn’t participate in the data collection have to be considered. Particularly, children whose parents did not complete the CHEQ survey were more vulnerable on the EDI domains than children on whose parents participated in the CHEQ (see Table S3). Previous research has shown that selection bias in such case leads to an underestimation of the association between the risk factor (i.e., high screen time) and outcome (i.e., vulnerability on EDI domains) [56]. Further, the CHEQ survey was only available in English and parents who were unable to read/understand English may not have participated. Hence, findings from this study may not be generalizable to other sub-populations of children in BC or Canada.

Limitations to the health behavior measures used in this study have to be noted. A common challenge with measuring screen time and other health behaviors is the subjectivity and potential recall bias of self-report measures provided by parents. Also, due to social desirability, some parents may under-report undesirable behaviors of their children. Lastly, even though the data on the CHEQ were collected six months prior to the EDI survey, temporality and causality cannot be established. It is possible that other underlying factors that were not measured contributed to both screen time and developmental health in this study.

An area of future research is to disentangle the specific contents of screen time and type of screen-based activities children in relation to developmental health. More studies are required to understand mechanisms, examine quality and content of screen use beyond quantity, and track children’s development and health longitudinally over time to provide more insight on supporting more specific policy recommendations for age appropriate screen use.

## Conclusions

Early experiences and behaviors play a crucial role in shaping a child’s developmental trajectories. Our study, drawing from a large-scale early childhood study, shows a negative association between longer screen time and young children’s developmental health in five core domains: physical, social, emotional, and cognitive development. These findings underscore the importance of limiting screen time in early childhood – as suggested by the 24-hour guidelines – and call for action to support the implementation of these guidelines in the Canadian context.

## Supplementary Information

**Additional file 1.**

## Data Availability

Data supporting the findings of this study are not publicly available for ethical and privacy reasons because the data pertain to individuals. In alignment with regulations for accessing population-level data in Canada, data can be accessed through Population Data BC’s Secure Research Environment cloud server and can be made available upon request and permission from the data steward through dataaccess@popdata.bc.ca.

## Abbreviations

STROBE: Strengthening the Reporting of Observational Studies in Epidemiology
BC: British Columbia
CHEQ: Childhood Experiences Questionnaire
EDI: Early Development Instrument
SD: Standard Deviation
GEE: Generalized Estimating Equations
OR: Odds Ratio
ANOVA: Analysis of Variance
MCAR: Missing completely at Random
MAR: Missing at random
MID: Multiple imputation then deletion
ICC: Intraclass Correlation
