# Supplementary material for: Screen time and developmental health: results from an early childhood study in Canada
Source: BMC Public Health. 2022 Feb 15;22:310. doi: 10.1186/s12889-022-12701-3 (PMC8845249; doi:10.1186/s12889-022-12701-3)
Supplement: Supplementary file 1 — Additional file 1. [file 12889_2022_12701_MOESM1_ESM.docx]

**Table S1**

*Sensitivity Analyses using Multivariable Logistic Regression Model Reporting Adjusted Odds Ratios and 95% Confidence Intervals for Relationship Between Screen Time and Vulnerability (Vulnerable vs. Not Vulnerable) on Five Developmental Health Domains Among Preschool-Aged Children in British Columbia, Canada, based on children with complete data, after deleting incomplete cases*

|  | Physical  health and wellbeing | Social  competence | Emotional maturity | Language and cognitive development | Communication skills |
| --- | --- | --- | --- | --- | --- |
|  | *OR*  (95% CI) | *OR*  (95% CI) | *OR*  (95% CI) | *OR*  (95% CI) | *OR*  (95% CI) |
| Screen time |  |  |  |  |  |
| > 1 hour | 1.39  (0.94 - 2.05) | 1.46*  (1.03 - 2.09) | 1.09  (0.79 - 1.51) | 1.56  (0.98 - 2.5) | 1.45  (0.95 - 2.22) |
| ≤ 1 hour | Ref |  |  |  |  |
| Sex |  |  |  |  |  |
| Male | 1.87***  (1.39 - 2.51) | 3.15***  (2.34 - 4.23) | 3.01  (2.3 - 3.93) | 1.55***  (1.11 - 2.17) | 2.14***  (1.55 - 2.97) |
| Female | Ref |  |  |  |  |
| Ethnicity |  |  |  |  |  |
| European origins | 1.13  (0.83 - 1.54) | 0.87  (0.65 - 1.15) | 1.09  (0.83 - 1.42) | 0.61  (0.43 - 0.86) | 0.47***  (0.34 - 0.65) |
| Not Euro. origins | Ref |  |  |  |  |
| Annual household income | |  |  |  |  |
| < $75,000 | 1.56*  (1.02 - 2.38) | 1.84**  (1.26 - 2.71) | 1.23  (0.86 - 1.76) | 1.93  (1.19 - 3.15) | 1.97**  (1.27 - 3.06) |
| ≥ $75,000 | Ref |  |  |  |  |
| Population Centre |  |  |  |  |  |
| Urban | 0.95  (0.67 - 1.35) | 1.11  (0.81 - 1.52) | 0.8  (0.59 - 1.08) | 0.81  (0.57 - 1.15) | 1.55*  (1.11 - 2.16) |
| Small to medium | Ref |  |  |  |  |
| Physical activity |  |  |  |  |  |
| Nonparticipant | 2.04***  (1.46 - 2.83) | 1.65**  (1.2 - 2.27) | 1.45  (1.06 - 1.97) | 1.92*  (1.32 - 2.78) | 2.04***  (1.43 - 2.92) |
| Participant | Ref |  |  |  |  |
| Sleep |  |  |  |  |  |
| < 10 hours | 1.39  (0.91 - 2.14) | 1.48  (0.99 – 2) | 1.7  (1.17 - 2.47) | 1.74**  (1.11 - 2.72) | 1.79**  (1.18 - 2.72) |
| ≥ 10 hours | Ref |  |  |  |  |
| Screen time X Income | 1.33  (0.75 - 2.35) | 1.02  (0.6 - 1.73) | 1.52  (0.92 - 2.52) | 1.03  (0.53 - 1.97) | 0.73  (0.39 - 1.35) |
|  | *N =* 2146 | *N =* 2144 | *N =* 2141 | *N* = 2134 | *N* = 2146 |
| *Notes.* Generalized Estimating Equations was used to account for clustering by schools. ^***^p < .001. ^**^p < .01. ^*^p < .05. *N* varies by missing values on developmental health domain | | | | | |

**Table S2**

*Sensitivity Analyses using Multivariable Linear Regression Model Reporting Unstandardized Beta Coefficients and 95% Confidence Intervals for Relationship Between Screen Time and Vulnerability (Vulnerable vs. Not Vulnerable) on Five Developmental Health Domains Among Preschool-Aged Children in British Columbia, Canada*

|  | Physical  health and wellbeing | Social  competence | Emotional maturity | Language and cognitive development | Communication skills |
| --- | --- | --- | --- | --- | --- |
|  | *B* (95% CI) | *B* (95% CI) | *B* (95% CI) | *B* (95% CI) | *B* (95% CI) |
| Screen time |  |  |  |  |  |
| > 1 hour | -0.18**  (-0.32 - -0.05) | -0.22*  (-0.4 - -0.04) | -0.16*  (-0.32 - 0.01) | -0.21*  (-0.38 - -0.05) | -0.2***  (-0.43 - 0.03) |
| ≤ 1 hour | Ref |  |  |  |  |
| Sex |  |  |  |  |  |
| Male | -0.36***  (-0.47 - -0.26) | -0.89***  (-1.03 - -0.76) | -0.86***  (-0.98 - -0.74) | -0.34***  (-0.46 - -0.21) | -0.75***  (-0.93 - -0.57) |
| Female | Ref |  |  |  |  |
| Ethnicity |  |  |  |  |  |
| European origins | -0.05  (-0.18 - 0.07) | 0.11  (-0.05 - 0.27) | 0.01 (-0.13 - 0.15) | 0.25**  (0.09 - 0.4) | 0.61***  (0.41 - 0.82) |
| Not Euro. origins | Ref |  |  |  |  |
| Annual household income | |  |  |  |  |
| < $75,000 | -0.24**  (-0.41 - -0.08) | -0.33**  (-0.54 - -0.12) | -0.25**  (-0.44 - -0.07) | -0.37***  (-0.57 - -0.17) | -0.54***  (-0.83 - -0.24) |
| ≥ $75,000 | Ref |  |  |  |  |
| Population Centre |  |  |  |  |  |
| Urban | 0.07  (-0.1 - 0.23) | -0.07  (-0.27 - 0.13) | 0.07  (-0.11 - 0.26) | 0.1  (-0.1 - 0.3) | -0.23  (-0.53 - 0.07) |
| Small to medium | Ref |  |  |  |  |
| Physical activity |  |  |  |  |  |
| Nonparticipant | -0.56***  (-0.71 - -0.42) | -0.58***  (-0.77 - -0.39) | -0.43***  (-0.6 - -0.26) | -0.61***  (-0.79 - -0.44) | -0.96***  (-1.21 - -0.71) |
| Participant | Ref |  |  |  |  |
| Sleep |  |  |  |  |  |
| < 10 hours | -0.36***  (-0.54 - -0.18) | -0.45***  (-0.69 - -0.21) | -0.37***  (-0.58 - -0.16) | -0.43***  (-0.65 - -0.21) | -0.8***  (-1.11 - -0.5) |
| ≥ 10 hours | Ref |  |  |  |  |
| Screen time X Income | -0.23  (-0.47 - 0.02) | -0.2  (-0.52 - 0.11) | -0.15  (-0.43 - 0.13) | -0.21  (-0.5 - 0.07) | -0.09  (-0.5 - 0.33) |
| *Notes.* *N* = 2,818. Generalized Estimating Equations was used to account for clustering by schools. The school-level variability on developmental health outcomes ranged from ICC = 0.12 to ICC = 0.14. Multiple imputation chained equation (MICE) approach was used to impute missing values. Multiple Imputation then Deletion (MID) was used to deal with missingness in dependent variables. ^***^p < .001. ^**^p < .01. ^*^p < .05. | | | | | |

**Table S3**

*Comparison of the proportion of child vulnerability on each EDI domain when EDI data were linked to CHEQ data in participating school districts, versus when EDI data were not linked*

|  | With CHEQ (% vuln) | Without CHEQ (% vuln) |
| --- | --- | --- |
| Physical health and wellbeing | 11.3 | 19.0 |
| Social competence | 13.4 | 18.9 |
| Emotional maturity | 15.2 | 18.7 |
| Language and cognitive development | 8.7 | 13.9 |
| Communication skills | 10.4 | 16.5 |
| Overall | 28.1 | 37.9 |
|  | *N* = 2931 | *N* = 1948 |
